# English learning burnout: Scale validation in the Chinese context

**DOI:** 10.3389/fpsyg.2022.1054356

**Published:** 2022-12-20

**Authors:** Honggang Liu, Yuchen Zhong

**Affiliations:** ^1^School of Foreign Languages, Soochow University, Suzhou, China; ^2^School of Foreign Languages, Northeast Normal University, Changchun, Jilin Province, China

**Keywords:** English learning burnout, construct, levels, exhaustion, demotivation, Chinese senior high school students

## Abstract

While there has been considerable research on learning burnout in educational psychology, it has received little attention in language education. To fill this gap, the present study aims at exploring the construct of English learning burnout (ELB) and examining the levels of this complex construct in a large sample of 1,213 Chinese senior high school students utilizing a 15-item *Senior High School English Learning Burnout Scale*. A two-dimensional structure of ELB comprising exhaustion and demotivation was extracted by exploratory factor analysis and validated by confirmatory factor analysis. A descriptive analysis showed low levels of global ELB and exhaustion and a moderate level of demotivation. The findings suggest that teachers should be mindful of fostering students’ self-regulated strategies to alleviate their ELB.

## Introduction

In contrast to the burgeoning research on positive psychological variables in language education, such as enjoyment, resilience and flow (e.g., Jin and Zhang, 2018; Dewaele J. et al., 2019; Liu and Song, 2021; Liu and Han, 2022), negative emotions, except for language learning anxiety, have received minimal research interest (Dewaele J. M. et al., 2019). Although English learning burnout (ELB) is among the critical factors of these negative emotions and it has detrimental effects on English learners (Jahedizadeh et al., 2015; Liu et al., 2021), ELB has been underexplored in the field of language education (Erakman and Mede, 2018). Limited studies were carried out to examine the inner structure and levels of ELB. However, due to the domain-and context-specific nature (Maslach et al., 2001; Schaufeli et al., 2009) of burnout in English learning, it seems possibly different from burnout in other subject learning and is worth investigating in various contexts.

In the English as a foreign language (EFL) context in China, English learners might be overloaded with English learning tasks and encounter rigid English learning requirements, examination-oriented English content and high expectations regarding their English learning achievement. Therefore, ELB as a negative emotional factor seems common in Chinese English classrooms and among Chinese English learners. For Chinese senior high school students especially, ELB could appear to be more intense and overwhelming because senior high school English learners would confront more demanding English learning tasks to improve their test scores under the excessive pressure due to the college entrance examination. Therefore, they would seem more likely to experience mental and physical exhaustion in English learning, detach themselves from English learning tasks and experience a decrease in their self-evaluation of their English learning efficacy.

Due to the above theoretical and practical purposes, the present study aims at unpacking the internal structure of burnout in senior high school English learning in the Chinese context through a survey administered to a large sample.

## Literature review

### Defining English learning burnout

ELB is “a negative chronic psychological and emotional state that students feel about their FL learning and FL class” (Li et al., 2021, p. 10). It was derived from job burnout (i.e., work burnout) in psychology and academic burnout (i.e., school burnout, student burnout or learning burnout) in general education. Almost all studies on ELB have defined it as a three-dimensional construct, comprising exhaustion, cynicism and reduced efficacy^1^ (Jahedizadeh et al., 2015; Erakman and Mede, 2018; Moghadam et al., 2020; Li et al., 2021; Liu et al., 2021), based on the validation of a trifactorial structure (i.e., exhaustion, cynicism and reduced efficacy) in research on work/job burnout and academic burnout (Malakh-Pines et al., 1981; Maslach and Jackson, 1982; Maslach et al., 2001; Schaufeli et al., 2002; Bresó et al., 2007; Schaufeli et al., 2009; Maslach and Leiter, 2016).

In ELB, exhaustion refers to the feeling of fatigue English learners experience in English learning activities. Cynicism denotes a detached and indifferent attitude toward English learning. Reduced efficacy refers to a decline in learners’ evaluation of their efficiency and ability to learn English (Yang, 2015; Li et al., 2021). ELB is a language-specific and context-related concept. Its development is closely associated with the purpose, content, method and environment of English learning (Li et al., 2021). Therefore, it is hypothesized that ELB may manifest in different forms when the context and participants change. Therefore, there is a need for further in-depth research to explore the internal structure of ELB.

### English learning burnout in language education

The fruitful achievements of research on job burnout and academic burnout in general education or psychology inspired the research on ELB in language education. However, ELB has not yet received significant research attention. The research topics of ELB cover explorations of the constructs of ELB and examinations of the levels of ELB subdimensions (e.g., Jahedizadeh et al., 2015; Erakman and Mede, 2018; Li et al., 2021) and its relationship with other factors (e.g., anxiety and mindfulness-based instruction; see Moghadam et al., 2020; Liu et al., 2021; Li, 2022).

### Exploration of the English learning burnout construct

In general, researchers have used surveys to study the internal structure of ELB. The *Maslach Burnout Inventory-Student Survey* (MBI-SS; Schaufeli et al., 2002) has been widely used to assess academic burnout due to its good reliability and validity. Researchers in language education have either employed the original version of MBI-SS (e.g., Jahedizadeh et al., 2015; Erakman and Mede, 2018; Moghadam et al., 2020) or adapted the wordings to better fit the English context (e.g., Liu et al., 2021). There was also an attempt to develop an English-specific instrument measuring learners’ ELB. For instance, Li et al. (2021) explored the ELB of 1,718 Chinese secondary school students and developed a 10-item *Maslach Burnout Inventory-English Student Survey* based on the original 15-item MBI-SS. These endeavors to work on the constructs of ELB yielded a similar finding – that is, a trifactorial structure comprising exhaustion, cynicism and reduced efficacy (e.g., Jahedizadeh et al., 2015; Erakman and Mede, 2018; Moghadam et al., 2020; Li et al., 2021; Liu et al., 2021). These constructs were defined by Maslach (1998) and Schaufeli et al. (2002).

### Examination of the levels of English learning burnout

Besides the ELB construct, researchers have explored students’ ELB levels. Our review of the limited literature on ELB revealed that scholars had detected the structure of ELB and tested the global and dimensional levels of ELB. For example, Erakman and Mede (2018) investigated Turkish English learners’ ELB levels. Among the three subdimensions, the levels of exhaustion and cynicism were high, while the level of reduced efficacy was low. Their results showed that the overall ELB status was medium. Liu et al. (2021) explored the relationship between self-oriented perfectionism and ELB under the effect of grit and language learning anxiety among 544 Chinese college students. Their findings evinced a low overall level of students’ ELB. Li et al. (2021) measured 1,718 Chinese senior high school students’ ELB using a 10-item *Maslach Burnout Inventory-English Student Survey*. They reported low overall and dimensional levels of ELB among Chinese English learners. Few studies have focused on English learners’ demographic factors and ELB status. For instance, Jahedizadeh et al. (2015) examined 250 Iranian English learners’ ELB to determine whether students’ educational levels (e.g., language institute and university) and gender influenced their ELB levels. Their results indicated that English learners from language institutes had higher overall and dimensional ELB levels than university students. They also found that male students had higher ELB levels than female students. Specifically, male students had significantly higher exhaustion and cynicism levels than female students.

In addition to research on the construct and levels of students’ ELB, there have been limited attempts to explore the relationship between ELB and other psychological factors, such as ELB and grit (Liu et al., 2021), and ELB and enjoyment (Li, 2022). Some studies also suggested practical solutions for alleviating students’ ELB, including weaponizing students with self-regulated learning strategies (SRLS). SRLS have been well-recognized as an effective way to remove negative English learning affective factors (Chow et al., 2018; Teng and Zhang, 2020). In the context of English education, self-regulatory English learners are supposed to be proactive in their English learning and they are equipped with the ability to manage, organize, control and evaluate their English learning process (Bown, 2006; Bown and White, 2010; Teng et al., 2020; Teng and Zhang, 2022). English learners could utilize these various strategies to control their driving forces, thinking processes, emotional states (Bown, 2006; Bown and White, 2010) and behavioral patterns (Bandura and Walters, 1977). Thus, English learners experiencing burnout may resort to self-regulated strategies, but this topic has remained unexplored in the ELB research.

As reviewed above, theoretically, abundant studies in psychology have confirmed the multidimensional burnout theory proposed by Maslach (1998). Yet some dispute is ongoing over the constructs of burnout, such as an exhaustion-centered (e.g., Kristensen et al., 2005; Shirom and Melamed, 2006) and two-dimensional structure of burnout (e.g., Halbesleben and Demerouti, 2005). The limited studies in language education seem to concur with the three-dimensional ELB structure comprising exhaustion, cynicism and reduced efficacy. However, the discrepant opinions on the burnout construct in psychology inspired us to think that burnout may have specific features and subdimensions in different domains. In the language education context, we wonder whether context-specific ELB would present a distinct structure rather than a three-dimensional one and whether the three-dimensional ELB construct would remain valid and robust among different research samples. Thus, this study aims at exploring the ELB construct among Chinese senior high school English learners. Practically, the common negative emotional factor of ELB has devastating ramifications for English learners (Jahedizadeh et al., 2015; Liu et al., 2021). Therefore, probing the levels of ELB could offer a general understanding of Chinese senior high school students’ ELB status and provide insight into ELB prevention and intervention. Therefore, the present study addresses the following questions:

RQ1. What are the reliability and validity of the *Senior High School English Learning Burnout Scale*?
RQ2. What are the levels of Chinese senior high school students’ ELB?

## Methods

### Participants

The participants of this study were 1,213 Chinese senior high school students. They were all native Chinese speakers taking compulsory English courses at school. These participants came from model schools^2^ (*N* = 991, 81.70%) and common schools (*N* = 222, 18.30%) in the northeast of China. There were 545 male (44.93%) and 668 female (55.07%) participants.

### Instrument

A 15-item *Senior High School English Learning Burnout Scale* was used to delve into the construct of ELB in the current study. This 6-point Likert scale is based on the MBI-SS developed by Schaufeli et al. (2002). The whole questionnaire consists of three sub-scales: exhaustion, cynicism and reduced efficacy^3^. All items were reworded to suit the English learning context when we translating them into Chinese for the final investigation.

### Data collection and analysis

The finalized scale was circulated to senior high school students *via* the survey data collection platform Wenjuanxing^4^ from December 2021 to January 2022. To explore the subdimensions of ELB, we performed the exploratory factor analysis (EFA) *via* SPSS 26.0 and the confirmatory factor analysis (CFA) *via* AMOS 24.0. The collected data were split into two parts for EFA (*N* = 621) and CFA (*N* = 592). Based on the data for CFA, the levels of students’ overall and dimensional ELB were analyzed.

## Results

### Exploratory factor analysis of English learning burnout

Before carrying out the EFA and CFA, we examined the normality of the data. The skewness and kurtosis statistics of each item (−2 to +2) showed a normal distribution of the collected data (Bryman and Cramer, 1999).

We adopted principal axis analysis in the EFA to extract factors and used the direct oblimin method to explore the internal structure of the data. Following the analyses of Ho (2006) and Hair et al. (2018), we deleted items with a factor load below 0.3 and those that were theoretically or logically inconsistent with other items in the same dimension. The test of Kaiser-Meyer-Olkin showed the data was very suitable for EFA (KMO = 0.896, *χ*^2^ = 6853.483, *df* = 45, *p* = 0.000; Wu, 2010). After deleting five items (items 11, 09, 15, 12, and 05 in order), two factors were extracted. Table 1 shows that the cumulative explanatory variance was 80.789%, far exceeding the generally accepted 55% cumulative explanatory variance (above 55%, the referential line for EFA; *cf.* Plonsky and Gonulal, 2015). We also obtained higher reliabilities of the two factors (0.957 and 0.954) and the whole scale (0.893). As Table 1 indicates, factor 1 consisted of cynicism (items 07 and 06) and reduced efficacy (items 10, 08, 13, and 14) in the original three-dimensional ELB structure (Schaufeli et al., 2002). Factor 2 was consistent with exhaustion (items 02, 03, 04 and 01) in Schaufeli et al. (2002). We further tested this bifactorial structure *via* CFA and discussed how they were named.

**Table 1:** Factor analysis of English learning burnout (pattern matrix).

Items	Factor 1 demotivation	Factor 2 exhaustion	Communalities
Q07 I have become less enthusiastic about my studies	0.917		0.846
Q06 I have become less interested in my studies since my enrollment at the university	0.907		0.832
Q10 During class I feel confident that I am effective in getting things done	0.902		0.809
Q08 I believe that I make an effective contribution to the classes that I attend	0.896		0.807
Q13 I can effectively solve the problems that arise in my studies	0.871		0.753
Q14 In my opinion, I am a good student	0.807		0.647
Q02 Studying or attending a class is really a strain for me		0.946	0.895
Q03 I feel burned out from my studies		0.936	0.878
Q04 I feel tired when I get up in the morning and I have to face another day at the university		0.92	0.842
Q01 I feel emotionally drained by my studies		0.876	0.771
Extraction Sums of Squared Loadings Cumulative %	50.086	80.789	—
Cronbach’s *α*	0.957	0.954	—

All the items in this table came from Schaufeli et al. (2002, pp. 477-478). “Studies” in this table were reworded into “English studies” in the scale for the current study. Items 08, 10, 13, and 14 were reversely expressed in Chinese in the scale for the final investigation.

### Confirmatory factor analysis of English learning burnout

**Figure 1:**
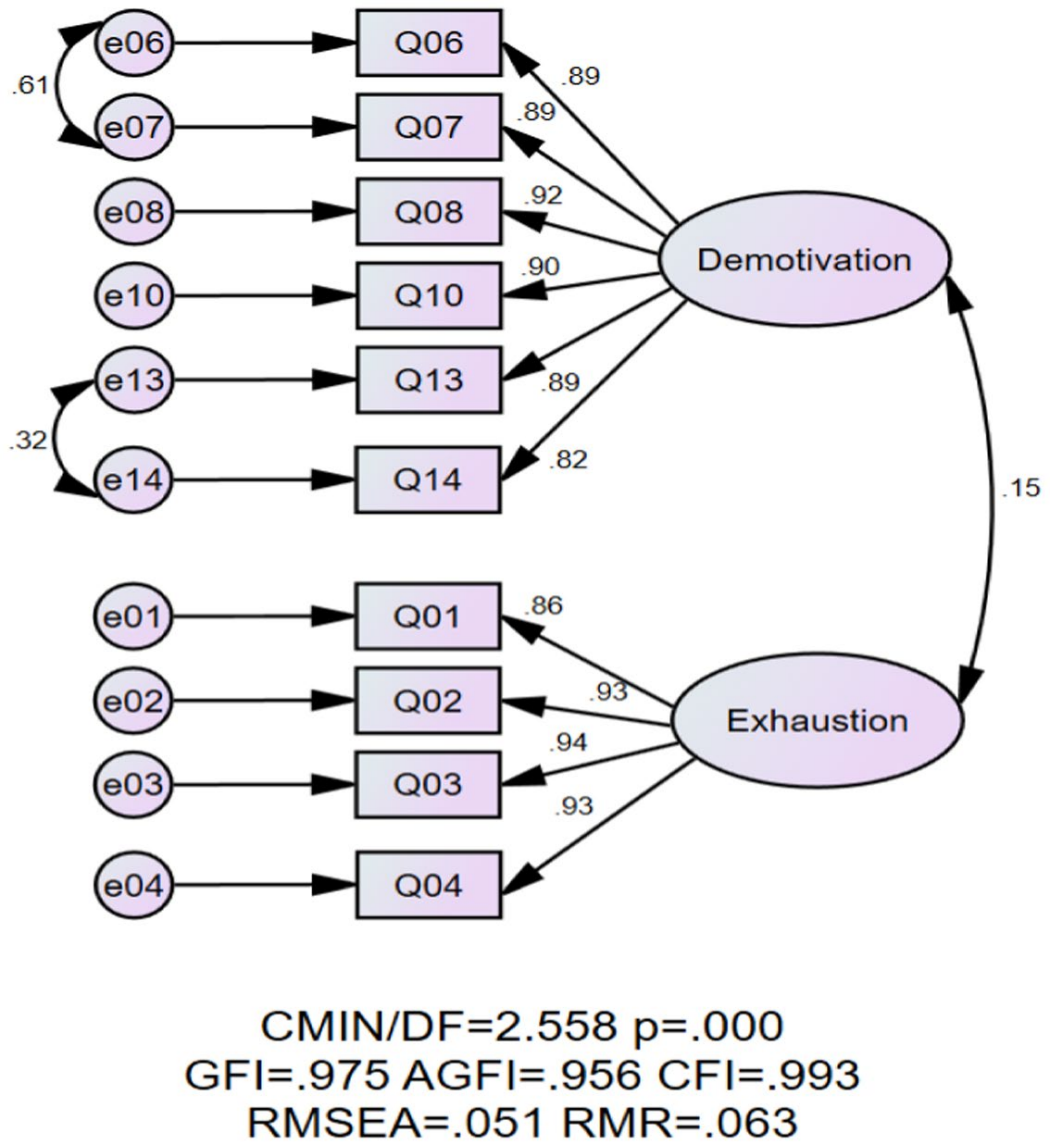
Graphical representation of the bifactorial model and factor loadings.

Two rounds of CFA were conducted to obtain an acceptable model. According to the CFA results, the bifactorial model with 10 items showed a good fit to the data (see Figure 1; Table 2). We established benchmarks for the indices assessing model fit (*χ*^2^/*df* ≤ 8; GFI ≥ 0.90; AGFI ≥ 0.90; CFI ≥ 0.90; RMSEA ≤ 0.08; RMR ≤ 0.10) specified by Kline (2016) and Wu (2010). As Table 2 displays, *χ*^2^/*df*, RMSEA and RMR were 2.558 (≤8), 0.051 (≤0.08) and 0.063 (≤0.10), respectively, and GFI, AGFI, and CFI were higher than 0.90 in the present model. All these data supported the bifactorial structure.

**Table 2:** Goodness of fit of the ELB scale with measurement models.

	*χ*^2^/df	Value of *p*	GFI	AGFI	CFI	RMSEA	RMR
Benchmark	≤8	≥0.05	≥0.90	≥0.90	≥0.90	≤0.08	≤0.10
Model 1	9.852	0.000	0.888	0.819	0.955	0.122	0.083
Model 2	3.879	0.000	0.961	0.935	0.986	0.070	0.070
Model final	2.558	0.000	0.975	0.956	0.993	0.051	0.063

Aside from the overall construct validity of the ELB scale, convergent validity and discriminant validity were considered in this study. Specifically, values of average variance extracted (AVE) were higher than 0.5 and values of composite reliability (CR) were higher than 0.7, revealing good convergent validity (Hair et al., 2018). As shown in Table 3, the results displayed good convergent validity of this model.

**Table 3:** Convergent validity and discriminant validity of each subscale.

Factor	Item number	Convergent validity			Discriminant validity (*r*)	
		Value of *p*	CR	AVE	Demotivation	Exhaustion
Demotivation	06	0.000	0.956	0.783	**0.885**	
	07	0.000				
	08	0.000				
	10	0.000				
	13	0.000				
	14	0.000				
Exhaustion	01	0.000	0.954	0.837	0.154**	0.915
	02	0.000				
	03	0.000				
	04	0.000				

**The bold number in discriminant validity represents the square root value of AVE.

Determining the discriminant validity of latent variables required comparing the values of AVE and correlation coefficients (Hair et al., 2018). As indicated in Table 3, the square root value of AVE of subscales was higher than its corresponding correlation coefficients (*R*^2^), suggesting good discriminant validity of each subscale.

Following the initial model testing, we assessed the measurement invariance of bifactorial ELB across gender using AMOS 24.0. Different structural models were tested through the following steps. In the first step, we conducted separate CFAs for male and female groups to look for the same patterns of structural fit indices. Next, we conducted multiple-group CFA (MG-CFA) to check whether the English burnout construct was invariant across male and female students. Equality constraints were included in each model. The models were assessed against less-constrained models with the following indices: configural invariance (M1), metric invariance (M2) and factor covariance invariance (M3). The values can be seen in Table 4. The CFA results showed an excellent fit for both male and female students. The goodness-of-fit indices were similar for both groups. In addition, the MG-CFA results exhibited configural, metric and factor covariance invariance of the bifactorial ELB model, as the chi-square differences (Δ*χ*^2^) between the models were non-significant (*p* > 0.05; Cheung and Rensvold, 2002).

**Table 4:** Fit indices for measurement invariance of the ELB model across gender.

Model	*χ* ^2^	*df*	*χ*^2^/df	GFI	AGFI	CFI	RMR	RMSEA	Δ*χ*^2^(Δdf)
Male	53.19*	32	1.662	0.964	0.938	0.993	0.073	0.049	—
Female	92.27*	32	2.883	0.949	0.912	0.983	0.073	0.078	—
M1	145.45*	64	2.273	0.956	0.924	0.988	0.073	0.046	—
M2	152.36*	72	2.116	0.954	0.930	0.988	0.087	0.043	6.91 (8)
M3	155.51*	75	2.074	0.953	0.931	0.988	0.136	0.043	3.15 (3)

*p < 0.05.

The EFA and CFA results led to the elimination of five items (items 05, 09, 11, 12, and 15) due to low factor loadings, and two dimensions emerged. Internal consistency reliability was measured using the Cronbach’s α coefficient. Alphas for the general ELB scale and its two dimensions were 0.893, 0.957, and 0.954, respectively. Additionally, the composite reliability and Cronbach’s α values were higher than 0.80, indicating the higher reliability of this scale (Hair et al., 2018).

Factor 1 includes two items (items 06 and 07) of the original cynicism dimension and four items (items 08, 10, 13, and 14) of reduced efficacy. All these items displayed the students’ demotivation, an emotion in the ‘negative process that reduces or diminishes a person’s motivation in relation to a behavioral intention or an ongoing action’ (Dörnyei and Ushioda, 2021, p. 140). Therefore, Factor 1 is named demotivation. Both external and internal factors possibly result in English learning demotivation (Gao et al., 2022; Gao and Liu, 2022). Demotivated learners experience low confidence caused by external factors, such as a change in learning environment (item 6) and possible highly challenging tasks in English class (item 10). They suffer from less enthusiasm toward English learning for internal factors, such as the incapacity to solve problems in learning English effectively (item 13) and negative self-evaluation (items 07, 08, and 14). Factor 2 contains four items of the original exhaustion dimension; therefore, this factor is named exhaustion. This result is consistent with numerous enquiries into job burnout in psychology. It reflects the core value and robustness of the exhaustion dimension of the burnout concept in different contexts, such as the workplace and language education.

The EFA and CFA results revealed ELB to be a two-dimensional concept including two subdimensions of demotivation (Factor 1) and exhaustion (Factor 2) in the context of Chinese senior high school English education. The two-dimensional ELB structure differed from the original three-dimensional structure of job burnout in psychology (Maslach, 1998) and foreign language learning burnout newly proposed in foreign language education (Li et al., 2021). We will discuss the reasoning behind the naming of these two factors in the Discussion part.

### Levels of Chinese senior high school students’ English learning burnout

Table 5 provides an overview of the overall and dimensional status of Chinese senior high school students’ ELB. According to the descriptive statistics, students’ overall ELB levels were low (*M* = 3.10, *SD* = 1.23). Regarding the dimensional status, the levels of demotivation and exhaustion were both low, but the demotivation level (*M* = 3.44, *SD* = 1.63) was higher than the exhaustion level (*M* = 2.61, *SD* = 1.53).

**Table 5:** Global and dimensional levels of students’ ELB (*N* = 592).

	Min	Max	*M*	SD
Exhaustion	1.00	6.00	2.61	1.53
Demotivation	1.00	6.00	3.44	1.63
Global ELB	1.00	6.00	3.10	1.23

## Discussion

### The bidimensional construct of English learning burnout

In the current study, Factor 1, demotivation, was a newly identified dimension including items from the widely accepted concepts of cynicism and professional efficacy. To some extent, this finding supports that burnout is a domain-dependent and context-related concept, which manifested its unique feature in this study when the subject matter was concerned. The primary reason might be that demotivation can share core values with the original cynicism and reduced efficacy dimensions. Regarding the definition, cynicism in the original three-dimensional ELB construct suggests students’ gradual detachment and indifference to English learning (Yang, 2015; Li et al., 2021). It is a negative process of students’ reduced interest and enthusiasm for English learning activities and tasks instead of actively and subjectively approaching English learning. Reduced efficacy focuses on the decrease in learners’ evaluation of their efficiency and ability in English learning (Yang, 2015; Li et al., 2021), which is also a negative process in which students become less confident in their own English learning effectiveness. Reduced efficacy contrasts with the increase in learners’ evaluation of their English learning efficacy.

It must be mentioned that the two dimensions of cynicism and reduced efficacy do not denote that students are neglectful and indifferent to or have a low sense of efficacy in English learning from the beginning. On the contrary, burnt out English learners’ cynicism and reduced efficacy are mainly caused by overloaded English learning tasks and activities. Students with ELB may feel physically and mentally exhausted, have the idea of escaping from English learning and rarely experience progress and achievements in learning English. In this regard, cynicism and reduced efficacy could be considered the behavioral intentions of English learners to detach from English learning under the influence of external and internal factors. Cynicism and reduced efficacy are also related to the ongoing actions of decreasing evaluation of English learning. This is consistent with the definition of demotivation in language education – namely, a detrimental process that lowers or weakens someone’s motivation regarding a behavioral aim or current activity for internal factors (Sakai and Kikuchi, 2009; Xaypanya et al., 2017; Gao et al., 2022) and external factors (Falout et al., 2009; Kikuchi, 2009, 2015; Kim et al., 2018; Gao et al., 2022).

In psychology, the initial exploration of job burnout was based on the dimension of exhaustion. Then in further investigations, the other two dimensions of cynicism and reduced efficacy gradually emerged (Maslach, 1998; Maslach et al., 2001). Therefore, exhaustion has always been considered the core of job burnout, which has been analyzed thoroughly and reported most widely (Maslach, 1998; Maslach et al., 2001; Maslach and Leiter, 2016). Some scholars have even directly equated the concept of burnout to the exhaustion dimension (Maslach, 1998), which showed the prominence and significance of exhaustion in the burnout conceptualization. In this study, the exhaustion dimension remained robust and stable after the EFA and CFA explorations of Chinese senior high school students’ ELB. This result indicates that the foundation of the concept of burnout lies in the exhaustion dimension – that is, physical and psychological fatigue under task overload.

### Levels of Chinese senior high school students’ English learning burnout

According to the descriptive statistics, Chinese senior high school students’ overall and dimensional ELB levels were low. This finding is consistent with Liu et al. (2021) and Li et al. (2021), whose studies also indicated low ELB among Chinese English learners. Students’ demotivation level was higher than their exhaustion level between the two subdimensions. In other words, Chinese senior high school English learners had mild ELB syndrome. They were more likely to decline in their English learning interest, passion, confidence and self-evaluation than to feel physical and psychological fatigue with English learning.

Whether it is job burnout in psychology or academic burnout in general education, the core of burnout is expressed as a response to chronic stress (Maslach, 1998; Maslach et al., 2001; Maroco and Campos, 2012). This chronic response is closely related to the ecological environment in which individuals live. Macroeducational policies concerning the English learning process, a classroom design that is directly associated with guidelines, English teachers’ instruction techniques and the creation of the teaching environment may all impact student ELB. Chinese English education has been controversial for a long time because of its test-oriented approach that overemphasizes grammar and skills and ignores students’ real English learning needs (Gao, 2008; Pan and Block, 2011; Ma and Bui, 2021; Zeng and Huang, 2021). However, several reform initiatives have been adopted at all levels of English education in China in recent years.

Thus, Chinese English education, including senior high school English education, has gradually altered to pursue a quality-oriented, communication-oriented and student-oriented approach (Yu and Wang, 2009; Zhang and Luo, 2022). From a macroscopic perspective, against the background of the new basic education curriculum reform in China^5^, cultivating students’ key competencies has become a new and permanent task for education in all subjects and at all academic levels. Thus, focusing on students’ key competencies, English education in Chinese senior high schools emphasizes reducing the burden on students and further improving the curriculum plan, curriculum standards and English teaching materials (Fang and Garland, 2014). In 2021, the General Office of the State Council issued the ‘Opinions on further reducing the burden of student homework and off-school training at the compulsory education stage’ – that is, the double reduction policy^6^. The double reduction policy has been gradually implemented in schools at all levels in China and has dramatically impacted teaching activities, including senior high school English education (Xue and Li, 2022). Under the guidance of the macro policies (e.g., the new curriculum reform and the double reduction policy), Chinese senior high school English teachers have also reformed their classroom teaching activities from the micro perspective. They have attempted to reduce students’ burden of homework and training outside of school, create a more relaxing and pleasant English classroom environment for students, improve classroom teaching efficiency and enhance students’ English learning outcomes (Zhang and Liu, 2014; Zhang and Luo, 2022). Therefore, in the context of reducing the burden on English learners and providing quality-oriented English education in China, senior high school students have faced less English learning burden, workload and stress in and outside English classrooms. Without excessive learning requirements and pressure, students are less likely to feel exhausted, demotivated and burnt out in their English learning. The reduced English learning burden of Chinese senior high school students, then, may not be sufficient to cause a moderate to high level of ELB.

In addition, the low ELB level of Chinese senior high school students may be related to the sampling of this study. About 80% of the participants were from model senior high schools. These students with higher English proficiency^7^ may be more confident in English learning and better able to independently use learning strategies to control their English learning. Therefore, they may not be easily affected by ELB.

The current study found students’ demotivation level to be higher than their exhaustion level, indicating that they might be more likely to experience reduced motivation in English learning, such as having declining English learning interest and self-confidence, than to feel physically and mentally exhausted in the process of learning English. Demotivation occurs in everyday English learning at different educational levels (e.g., high school and university levels; see Hassaskhah et al., 2015; Xaypanya et al., 2017; Gao et al., 2022; Gao and Liu, 2022). It could be caused by external factors, such as the incompetence of language teachers and inappropriate teaching materials or methods (Kikuchi, 2009; Karaca and Inan, 2020; Wang and Guan, 2020; Wu et al., 2020), and internal factors, such as prior language learning experience, interest, anxiety and negative attitudes (Kim, 2011; Shin-Hye, 2015; Xaypanya et al., 2017; Wang and Guan, 2020; Wu et al., 2020). These demotivators could erode students’ confidence and make them gradually lose their enthusiasm for and interest in learning English. Though the macro and micro environments have become better for students to pursue healthy psychological and academic development, the internal and external demotivators may remain. Students would continue to face difficulties and challenges in learning English. This partially explains why Chinese senior high school students demotivation level was higher than that of exhaustion.

## Conclusion and implications

The current investigation examined a bifactorial ELB (i.e., exhaustion and demotivation), which verified the multidimensional nature of burnout as a psychological variable in human learning and further confirmed that burnout is a domain-specific, contextualized construct. Exhaustion as a general psychological trait in English learning supported the central status of the exhaustion dimension in burnout (Maslach, 1998). This revealed the universality of exhaustion across domains and cultural contexts. The new dimension, demotivation, displayed the specialty of English learning burnout; that is, loss of interest or enthusiasm and less confidence in learning English were central demotivators to ELB. This two-dimensional construct of ELB motivated us to re-examine the concept of burnout in language education. We defined ELB as a negative, complex psychological state (Maslach et al., 2001; Maslach and Leiter, 2016) where learners feel demotivated by their confidence and interest and even exhausted regarding English learning. The current research, on one hand, answers the call for exploring the domain feature of burnout (Maslach et al., 2001; Schaufeli et al., 2009) and provides new evidence for burnout research in general; on the other hand, it has moved ELB research forward with its constructed bifactorial dimension.

The current study has pedagogical implications for future research. ELB commonly occurred among students irrespective of whether they came from a model or common school. Although Chinese senior high school students showed a relatively low level of ELB, English teachers and students should still not drop their guard. Instead, precautions and interventions must be considered because ELB has damaging impacts on students, such as reduced energy, lowered productivity and adverse learning outcomes (Jahedizadeh, 2015; Erakman and Mede, 2018; Moghadam, 2020). An effective approach for English teachers is to organize self-regulated strategy training (SRST) to enhance students’ emotional capacity in English learning. Self-regulation refers to exerting control over one’s driving forces, thinking processes, emotional states and behavioral patterns (Bandura and Walters, 1977). Self-regulatory students are able to manage and control their emotions, thoughts and behaviors to deal with challenging circumstances and EFL learning settings. Moreover, self-regulation incorporates cognitive, emotional and motivational elements (Paris and Paris, 2001). Therefore, self-regulatory students are expected to foster positive emotional factors and mitigate challenging learning affect (Tran et al., 2013). It is suggested that SRST be integrated into daily English teaching. In delivering the SRST, English teachers should firstly understand the general situation of students’ use of self-regulation strategies and then clarify to students the significance of SRST. They are advised to exemplify the self-regulation strategies (e.g., cognitive and motivational regulation strategies; see Wolters, 1999; Teng et al., 2020; Teng and Zhang, 2020) for the students in imparting their English knowledge. After the training, English teachers also need to track, monitor and evaluate students’ use of self-regulation strategies over time to promote the students’ development of effective self-regulation strategies. The SRST may assist students in preventing or reducing the status and harmful effects of ELB.

The present study also has some limitations and offers recommendations for future research. First, concerning the sampling, though the study was based on a large sample, we suggest that there should be a greater focus on the ELB of learners of different ages, such as junior high school students (13–16 years old) and university students (over 19 years old). The sampling should be balanced; for example, the samples from both model and common schools should be similar in size. Extending the sample types will also be better for validating the ELB scale of the current study. Since the focus of the current study was on exploring the reliability and validity of the ELB scale and examining the levels of students’ ELB, less attention was paid to the dynamics of ELB and its two internal factors (demotivation and exhaustion). As reviewed, exhaustion has been widely researched in psychology. However, there was less evidence relating to demotivation as a newly found dimension in ELB. Studies have shown that demotivation in English learning is dynamic (Dörnyei and Csizér, 2002; Song and Kim, 2017; Pack et al., 2022), and it is worth investigating the dynamics of demotivation *via* longitudinal quantitative or qualitative approaches in future studies.

In addition, a cross-country survey involving learners from different social and cultural contexts is a promising future direction for studying ELB. It will be beneficial to see the diversity of ELB and enrich the findings of ELB research. As reviewed and suggested in our study, SRST is effective in helping learners bounce back to normal from ELB. Therefore, experimental studies or action research should be conducted to explore how SRST can be trained to enhance students’ confidence and strategic English learning abilities. Future studies should also concentrate on the relationship between ELB and other psychological factors, such as emotions, SRST and motivation.

## Data availability statement

The original contributions presented in the study are included in the article/supplementary material, further inquiries can be directed to the corresponding author.

## Ethics statement

The studies involving human participants were reviewed and approved by School of Foreign Languages, Soochow University. The patients/participants provided their written informed consent to participate in this study.

## Author contributions

HL: conceptualization, data collection, data analysis, revision, supervision, and funding. YZ: conceptualization, data analysis, writing, and revision. All authors contributed to the article and approved the submitted version.

## Funding

This paper was supported by the Project of Discipline Innovation and Advancement (PODIA) – Foreign Language Education Studies at Beijing Foreign Studies University (grant number: 2020SYLZDXM011).

## Conflict of interest

The authors declare that the research was conducted in the absence of any commercial or financial relationships that could be construed as a potential conflict of interest.

## Publisher’s note

All claims expressed in this article are solely those of the authors and do not necessarily represent those of their affiliated organizations, or those of the publisher, the editors and the reviewers. Any product that may be evaluated in this article, or claim that may be made by its manufacturer, is not guaranteed or endorsed by the publisher.

## Footnotes

^1^In some studies, the term reduced efficacy was used interchangeably with professional efficacy or efficacy. Reduced efficacy was named for negative wordings of items (e.g., Li et al., 2021) or reversely scored positive (e.g., Schaufeli et al., 2002).

^2^In the basic education system in China, there are two types of senior high schools – namely, model senior high schools and common senior high schools – differentiated by several criteria (e.g., the number and quality of teaching equipment, number of classes and educational background of teachers) in a dynamic evaluation system. Model senior high schools admit students who achieve higher global marks than those recruited by the common senior high schools.

^3^We thoughtfully consider the doubt that the third dimension of Professional Efficacy was inconsistent (Bresó et al., 2007; Schaufeli and Salanova, 2007) and reported relatively lower reliability than the other two dimensions (Schaufeli and Salanova, 2007; Li et al., 2021). Meanwhile, in Schaufeli et al. (2002), all the items in the reduced efficacy dimension were positively stated, such as ‘I feel stimulated when I achieve my study goals’, but reversely scored for data consistency in the statistical analysis. Some studies (e.g., Jahedizadeh et al., 2015; Li et al., 2021) rephrased the items in the efficacy dimension in a reverse way to obtain direct responses regarding this negative emotion, which may effectively alleviate the above doubt. Therefore, in the final questionnaire, we reversely worded all the items (Q08, 09, 10, 13, 14, 15) in Schaufeli et al.’s (2002) efficacy/professional efficacy dimension.

^4^ https://www.wjx.cn/

^5^China’s new round of basic education courses was officially launched in 1999. From July 2000 to February 2001, the development team prepared the first draft of curriculum standards on the basis of careful research. In March, the Department of Basic Education of the Ministry of Education solicited opinions from educators, experts and scholars in nine areas. Further, it amended the standards for curricula of various disciplines. In July, the Ministry of Education promulgated the ‘Outline of Basic Education Course Reform (Trial)’. http://www.moe.gov.cn/jyb_xwfb/s5147/201909/t20190926_401046.html.

^6^The Opinions on Further Reducing the Burden of Homework and Off-Campus Training for Students at the Compulsory Education Stage aims to further reduce the burden of homework and off-campus training for students at the compulsory education stage. It was considered and adopted by the 19th meeting of the Central Committee for Comprehensively Deepening Reform on 21 May 2021. http://www.moe.gov.cn/jyb_xxgk/moe_1777/moe_1778/202107/t20210724_546576.html.

^7^The results of an independent-samples T-test indicated that there existed a significant difference between model and common school students (*t*(586) = 3.43, *p* < 0.01) regarding the standardized students’ mid-term English tests. Model school students obtained higher standardized scores (*M* = 0.06, *SD* = 1.05) than common school students (*M* = −0.31, *SD* = 0.66).
